# PrEP in India's HIV Prevention Policy in the Era of Social Media and Sex Positivity

**DOI:** 10.5195/cajgh.2020.407

**Published:** 2020-03-31

**Authors:** Anindya Kar, Dinesh Bhugra, Shuvankar Mukherjee, Agnibho Mondal, Aaditya Suresh Kumar

**Affiliations:** 1 Department of Psychiatry, Advanced Neuropsychiatry Institute, Kolkata, India; 2 Health Service and Population Research Department, Institute of Psychiatry, Psychology & Neuroscience, King's College London, London, UK; 3 Department of Community Medicine, Calcutta National Medical College, Kolkata, India; 4 Department of Tropical Medicine, School of Tropical Medicine, Kolkata, India; 5 Department of Neurology, Institute of Neurosciences, Kolkata, India

**Keywords:** HIV, PrEP, Social Media, India, Sex Positivity

## Abstract

**Introduction:**

The global revolution of online social media and connectivity had a tremendous effect on sexual behavior in both developed and developing countries. This global change is influencing the societal structure and existing social principles. Moreover, it has a significant impact on the epidemiology of different infectious diseases, especially HIV.

**Discussion:**

India is one of the most diverse democratic countries that has undergone a social-cultural transition in the last decade. However, having the second-highest HIV infection rate in the world, India does not have any other new prevention tools in their national HIV prevention strategy. Pre-Exposure Prophylaxis (PrEP), a boon of HIV prevention widely used in different countries, is still not implemented in India. The concept of “Digital India” by the Government of India is giving wide access of internet to the people of India. Furthermore, people are exposed to social media, and that is impacting their sex seeking behavior. Interestingly, recent legal changes in India promotes sex positivity. It also calls for introspection on existing HIV preventive strategies.

**Conclusions:**

Given the current scenario of PrEP and other existing preventive measurements of HIV, further research is needed to determine the acceptance and efficacy of PrEP and improve engagement in care for individuals in India. Various international studies recommend effective implication of PrEP to reduce the rate and economic burden of HIV infection.

India's socio-cultural transition in the last decade has been impactful on the demography of HIV^1^. The recent explosion in social media usage and the Digital India campaign are remarkably changing human interaction on many levels. The giants of social media like Facebook and Twitter, along with dating apps like Tinder and Grindr, are not restricted to a particular group of people in India but cater to wide and diverse populations. This heterogeneous distribution of social media users has an impact on sexual behavior and its risks. Furthermore, it appears that the incidence and prevalence of HIV varies among people of different cultural backgrounds due to an interplay of psychological and social factors. The provision of healthcare in any democratic country, such as India, is influenced by the demands of its people of many different cultural identities and relies on social, political and economic factors to achieve an optimal prevention policy. The recent changes in HIV prevention policy of India where every individual with HIV positive status are being treated with free antiretroviral therapy (ART) irrespective of their CD4 count are definitely^1^ benefitting the people living with HIV (PLHIV). However, HIV infection is a significant public health burden in India, and Pre-Exposure Prophylaxis (PrEP) is still an alien concept. The global sexual renaissance^2^ has changed societal attitude towards consensual online sex. In India, online dating applications and websites are also widely used, and the pattern of sex-seeking behavior has drastically changed in the last few years^3^. Policy makers need to take into consideration behavioral responses to changes in the cost of disease and implement strategies that are holistic and long-sighted. This paper will review these changes and how they impinge upon and, by so doing, help the policy makers and clinicians to identify the need of PrEP and address these issues in a culturally sensitive way.

## Discussion

### Global perspective of PrEP

Behavioral interventions and barrier methods, such as the use of condoms, slowed down the HIV epidemic in past^4^, but as long as no vaccine is available, new intervention strategies are still urgently needed. The two drug fixed-dose combination therapy with emtricitabine/tenofovir disoproxil fumarate (FTC/TDF) became the first to be used as PrEP, known as Truvada, and was approved by USA Food and Drug Administration (FDA) in July 2012 for high risk groups^5^. Both TDF and FTC are nucleos(t)ide analogue reverse transcriptase inhibitors (NRTIs). They have longer half-lives, which allow for less frequent dosing^6^. In fact, the half-lives of TDF/FTC are the longest for the NRTI class, a potentially favorable pharmacological characteristic for PrEP from an adherence perspective^3^. Prior to the FDA licensure there was a series of meta-analyses of studies on PrEP that had been done on high risk groups, i.e. those who are vulnerable to HIV infections. A sub-analysis of the men who have sex with men (MSM) study (known as the Pre-Exposure Prophylaxis Initiative [iPrEX]) showed that with optimal adherence, efficacy was more than 90%, and incidence of HIV infection was reduced by 92%^7^,^8^. Other trials, including one among serodiscordant heterosexual couples in Kenya and Uganda and another among sexually active young men and women in Botswana, have also demonstrated promising results for the use^9^ of PrEP as a prevention strategy. As clinical trials continue to establish efficacy, researchers have been increasingly interested in awareness and acceptability among potential candidates of PrEP. Studies have found that overall knowledge of PrEP is low to modest, with concerns relating to potential side effects, costs, drug resistance and accessibility^10^. On a recent study amongst HIV-negative individuals, some men suggested that the reason for the likely future adoption of PrEP was the opportunity to engage in sex without condom use, either with their serodiscordant partner or casual partners. Other participants equated PrEP adoption with greater sexual freedom^11^.

In July 2014, the World Health Organization (WHO) suggested that all MSM should consider taking PrEP in conjunction with other risk reduction strategies^12^. Countries like South Africa, Australia, United Kingdom, Germany and other European countries have considered PrEP as an emerging strategy and an important addition to the toolbox of HIV prevention^13^. Understanding the cultural context of PrEP provision is vital for implementation, with factors such as sexual practice, age and gender playing important roles in HIV acquisition risk and acceptability of interventions. Pressure has increased globally for countries to submit to regulatory authorities and include PrEP in national policies.

### HIV in India-Current scenario and challenges

**Figure 1.**
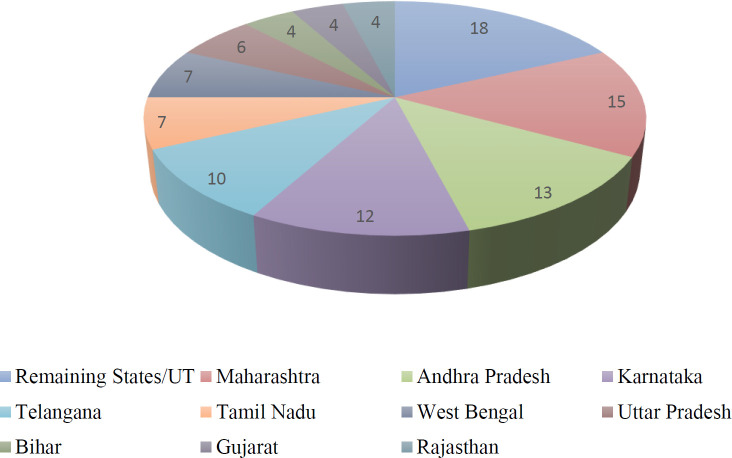
People Living with HIV, Percent Distribution Among States, 2017

Source: National AIDS Control Organization of India, 2017

The most prevalent mode of HIV transmission in India is sexual route^14^. According to the recent data of 2018, the new HIV infections increased to 88,000 from 80,000 and AIDS-related new deaths increased to 69,000 from 62,000 in India^15^. The UNAIDS data suggests almost 79% of PLHIV are aware of their status and on effective ART. The treatment efforts, primarily through ART, helped to manage their infection. Most importantly, the introduction of Indian government's ‘test-and-treat’ policy, irrespective of the CD4 counts and clinical stage of the disease, catalyzed the process of “Treatment as Prevention” (TasP)^11^ Moreover, the rate of new infections and deaths is not falling rapidly enough in meeting the 90–90–90 ambitious treatment target to help end the AIDS epidemic given by the Joint United Nations Programme on HIV/AIDS (UNAIDS)^16^. The state wise prevalence of HIV infections in India given by National AIDS Control Organization (NACO) in 2017 is elaborated in Table 1 and Figure 1. A recent study says PrEP with a biannual testing program has the potential to improve average per-person survival by nearly one year and block more than 270,000 HIV transmissions in India^17^. PrEP drug costs^18^ are lower than HIV treatment costs, both per-dose and for the duration of use. Moreover, PrEP is prescribed to be taken consistently, but only when someone is at heightened risk of HIV, whereas, should someone acquire HIV, they will need to be on ART for their entire life in order to stay healthy^18^,^19^. In May 2018, drug maker Cipla received regulatory approval in India to sell its version of Truvada as Tenvir EM^20^. However, this highly expensive monthly drug remains out of reach for many people who want and need it. There are no studies from India that can establish the efficacy or acceptance of PrEP in India. The only trial that has ever been done on PrEP in India focused on female sex workers, led by The Sonagachi Project in Kolkata^15^. The trial was designed to assess PrEP feasibility in that particular population but did not provide sufficient evidence to recommend that PrEP be made routinely available by the National or State AIDS Control Programs for high risk individuals.

**Table 1.** States with Adult (15–49 years) HIV Prevalence about the National Average, 2017

Region	Adult HIV Prevalence (%)
**India**	**0.22**
Mizoram	2.04
Manipur	1.43
Nagaland	1.15
Telangana	0.7
Andhra Pradesh	0.63
Karnataka	0.47
Goa	0.42
Maharashtra	0.33
Delhi	0.3
Tamil Nadu	0.22

### Social media and the change in sex-seeking behavior

With the development of the Internet and mobile technology, the ways of socializing and seeking sexual partners have changed dramatically^21^. Mobile online dating applications like Grindr and Tinder along with other social media sites are widely used to seek potential sexual partners among both heterosexual and homosexual people^22^. Seal et al. suggest that because the Internet can accommodate a variety of sexual expressions, as well as allow anonymity for the user, it provides an ideal environment to explore sexuality^23^. Online partner seeking also allows people to more precisely delineate specific partner characteristics, such as HIV serostatus or a desire to engage in certain types of sexual behaviours^20^. However, there is no literature on sex-seeking behavior in India, especially on the use of social media. Most of the current literature on the use of the social media has focused on MSM. In contrast, relatively little research, especially qualitative research, has focused on heterosexual individuals. This is potentially due to the large burden that HIV infection has on MSM populations or that MSM use the Internet more actively to find sex than heterosexual men and women^24^. It was estimated that in the beginning of 2018, there were almost 3 million users on one of the most popular heterosexual dating apps in India^25^. On the other hand, various gay dating apps also have a significant number of users, one example being Grindr, which boasts around 4.5 million users worldwide as of 2020^26^. Gay dating apps often give reminders of HIV testing, ask if the person is on PrEP and whether the person has an undetectable viral load if the person is seropositive^27^. These dating apps also spread the message that ‘undetectable equals untransmittable’^28^. Undetectable equals untransmittable is the message of the UNAIDS campaign that shows the evidence demonstrating that HIV treatment is highly effective in reducing the transmission of HIV^29^,^30^.

Apart from this, Facebook is also used for no strings attached sexual encounters^31^. Another popular application, Instagram, is also quite popular among the youth to find casual partners for physical intimacy^32^. There were around 201 million and 35 million Indian users on Facebook and Instagram respectively in 2017^33^.

### Sex positivity and legal changes

Sex positivity is defined as “a positive attitude to sexual activity that is seen as a healthy relationship and form of self-expression”^34^. Sexual pleasure is a valuable attribute in itself even without the context of marriage, procreation or an intimate relationship. It is, in itself, an important and positive part of human existence. “Hookups” or uncommitted sexual encounters, are becoming progressively more engrained in popular culture, reflecting both evolved sexual predilections and changing social and sexual scripts^35^. This particular trend has been catalyzed by emerging online dating apps coming into the mainstream and challenged the existing ideas of monogamous relationships, prioritizing consensual sex^36^.

Social change influences laws^37^,^38^. In 2014, the Supreme Court of India recognized the transgender persons as members of a third gender alongside male and female^39^. In January of 2018, the Supreme Court in its judgment on privacy, said that Right to Privacy and the protection of sexual orientation lie at the core of the fundamental rights guaranteed by Articles 14, 15 and 21 of the Indian Constitution^40^. On September 06, 2018, in a historic judgment, the Supreme Court of India removed consensual adult sex as a crime under section 377, saying sexual orientation is natural and people have no control over it^41^. Before this, the law particularly affected the Lesbian, Gay, Bisexual, Transgender (LGBT) community and violated the right to form association under Article 19 of the Indian Constitution^42^. On September 27, 2018, the apex court also ruled that adultery is no longer a crime under law, recognizing that the colonial-era law was unconstitutional and had some gender based discriminatory elements^43^.

### Empowering with PrEP

PrEP empowers the susceptible people including women to HIV infection^44^. PrEP also reduces the stigma of HIV infection and hence acts as an emancipator of people living with HIV by reducing the risk of acquiring HIV infection amongst serodiscordant couples^39^. PrEP is considered as an option for HIV-negative women who want to have a baby with their partner living with HIV^45^. It is also considered as a mature and empowering approach for a person who is in a relationship with a partner who is not willing to use preventive measures. Furthermore, it is a valuable method that allows women to protect themselves from HIV infection without dependence on their male sex partners to use condoms.

On the other hand, there is a wide range of stigma and stereotyping regarding certain sexual positions within the gay community^46^. The bottom, or receptive partner during sexual intercourse, is 13 times more likely to get infected with HIV than the top, who is the insertive partner^47^. Furthermore, bottom shaming is highly prevalent due to the gender roles related stigma, as the receptive partner is associated with femininity^48^. PrEP is the first opportunity bottoms have ever had to be in full control of their risk of HIV transmission. Moreover, it allows both partners, regardless of sexual position, to be responsible for their own protection^49^. In a country like India, where almost 90%^50^ of transgender people are involved in sex work, PrEP, if implemented, will be added to the arsenal of measures they can employ to protect themselves^51^.

## Recommendations

Implementation of PrEP is a long time constructive work. A few recommendations that can be considered are

- Policy makers should be sensitive to cultural backgrounds and aware of social changes.
- There should be further studies on acceptance, efficacy and side effects of PrEP in India.
- The medical curriculum needs to be gender sensitive and it is important to discourage moral judgement in clinical practices. The new updates of HIV medicine like effectiveness of ART to reduce transmissibility, usefulness of PrEP, etc. should be taught to the primary health care physicians.
- The first step in implementing PrEP is identifying persons at high risk of HIV acquisition. However, identifying such persons can be challenging due to perceived fear of stigma and social discrimination. The lack of a trusting relationship between the patient and the clinician may also make it harder. Hence it is important that clinicians routinely take a sexual and injection drug use history for all their patients in an open and nonjudgmental manner.

PrEP is only fully effective when it is adhered to exactly as prescribed. Furthermore, it does not protect against other sexually transmitted infections (STIs). Hence, it needs to be delivered as part of a comprehensive package of HIV/STI prevention services based on an individual's circumstances.
